# Assessment of Selected Biochemical Parameters of the Renin–Angiotensin–Aldosterone System in Repeat Convalescent Plasma Donors in the Context of Long-Term Changes Following SARS-CoV-2 Infection

**DOI:** 10.3390/jcm14144910

**Published:** 2025-07-10

**Authors:** Marta Stanek, Dorota Diakowska, Krzysztof Kaliszewski, Anna Leśków

**Affiliations:** 1 Regional Center of Transfusion Medicine and Blood Bank, 50-345 Wrocław, Poland; 2 Division of Medical Biology, Faculty of Nursing and Midwifery, Wroclaw Medical University, 50-368 Wrocław, Poland; 3 Department of General Surgery, University Centre of General and Oncological Surgery, Wroclaw Medical University, 50-556 Wrocław, Poland; krzysztof.kaliszewski@umw.edu.pl

**Keywords:** blood donation, COVID-19, renin–angiotensin–aldosterone system, SARS-CoV-2

## Abstract

**Background:** SARS-CoV-2 infection has been associated with long-term health consequences, including dysregulation of the renin–angiotensin–aldosterone system (RAAS). This study aimed to evaluate long-term changes in selected RAAS-related biochemical parameters in repeat convalescent plasma donors, focusing on enzymes and peptides involved in vascular regulation and inflammation. **Methods:** Thirty repeat convalescent plasma donors were enrolled, each providing four serum samples at defined time points post-infection. Samples were collected during Period 1 (≤60 days), Period 2 (61–90 days), Period 3 (91–120 days), and Period 4 (>120 days) after confirmed SARS-CoV-2 infection. The analyzed parameters included angiotensin I (Ang I), angiotensin II (Ang II), angiotensin 1–7 (Ang 1–7), angiotensin 1–9 (Ang 1–9), ACE, ACE2, ADAM10, and ADAM17. Concentrations were determined using ELISA assays. The control group consisted of pre-pandemic serum samples from healthy individuals. **Results:** An initial post-infection increase was observed in most parameters, particularly in Period 1. Over time, levels of several markers declined, yet Ang 1–7 and Ang 1–9 remained elevated compared to controls even beyond 120 days. Significant correlations (*p* < 0.05) were found between ADAM10, ADAM17, and angiotensin peptides, suggesting prolonged RAAS modulation. Metalloproteinases were notably elevated early after infection, potentially contributing to inflammatory and cardiovascular responses. **Conclusions:** The findings indicate a transient but measurable biochemical response of the RAAS following SARS-CoV-2 infection, with most parameters normalizing after 120 days. However, the sustained elevation of certain markers suggests a potential long-term impact on vascular homeostasis, warranting further investigation.

## 1. Introduction

In the course of global studies conducted since December 2019, in connection with the COVID-19 pandemic caused by infection with the SARS-CoV-2 virus, it has been shown that this virus enters the host cell by binding to the transmembrane part of angiotensin-converting enzyme II (ACE2) [1,2], which is part of the extended pathway of activation of the renin–angiotensin–aldosterone system (RAAS).

The RAAS plays an important role in the regulation of the water and electrolyte balance of the human body. It is also a key regulator of blood pressure through mechanisms involving vasoconstriction and modulation of sodium and water retention [3]. There is a classic pathway of system activation (angiotensin 1 is converted to angiotensin II by angiotensin I-converting enzyme, which, by acting on the angiotensin type I receptor, activates vasoconstriction, increases blood pressure and aldosterone secretion, intensifies cell proliferation and fibrosis processes, and enhances the inflammatory response and prothrombotic effects) and an extended pathway (angiotensin II-converting enzyme converts angiotensin I to angiotensin 1–9 and angiotensin II to angiotensin 1–7, which, by binding to the Mas receptor, cause anti-inflammatory, vasodilatory, and antithrombotic effects). In addition, angiotensin II has a hydrolyzing effect on the proteins of the apelinergic system (including apelin and elabela peptide), contributing to the development of circulatory system diseases, including the progression of atherosclerosis and hypertension [3,4,5,6,7].

It has been shown that viral diseases can cause an increase in the expression of the ACE2 enzyme, as well as an increase in the activity of ADAM-type metalloproteinases [8]. ADAM17 and ADAM10 have the ability to cleave proteins, including ACE2 associated with the cell membrane (mACE2), thereby increasing the level of its soluble form in plasma (sACE2). This reduces the infectivity of SARS-CoV-2 by removing potential cell entry sites. However, studies have indicated that sACE2 can form complexes with SARS-CoV-2 virions, which are thus transported to tissues, increasing organ failure [6,9,10]. While the available literature contains many publications on the concentration of converting enzymes or angiotensins themselves (Ang I, Ang II) in people infected with SARS-CoV-2 [9,11], there is a lack of information on the remaining important components of the RAAS, especially after a long time has passed since infection.

Determining the level of changes in these parameters in participants after SARS-CoV-2 infection, compared to samples obtained from people who have never been infected with SARS-CoV-2, is an important scientific consideration that allows for determining the pathophysiological impact of infection on the human body and may enable prediction of pathological changes in the future, including in the context of the impact on the progression of circulatory system diseases, or the development of water–electrolyte disorders and coagulation system dysfunctions. Importantly, the available literature is based on the measurement of the above-mentioned compounds in material collected from people currently ill with COVID-19 or up to 7 days after falling ill [12,13], meaning in the short period of time that has passed since the virus entered the host cells. Therefore, the long-term consequences of infection are unknown, including whether the pathological condition persists despite the cessation of active infection. Given the time that has passed since the onset of the COVID-19 pandemic, as well as the abundance of clinical and basic research focused on the mechanisms and clinical manifestations observed in patients during the course of active disease, there is an increasing need to investigate the long-term consequences and underlying mechanisms of SARS-CoV-2 infection—collectively referred to as long COVID or post-COVID syndrome. Among the symptoms and pathological features currently associated with long COVID are immune dysregulation [14], complement system dysfunction [15], thrombosis [16], dysbiosis [17], and viral persistence [18].

The aim of this study was to evaluate the concentrations of selected components of RAAS in the serum of repeat blood donors who had recovered from SARS-CoV-2 infection and to assess potential long-term changes associated with the infection. The obtained results were compared with pre-pandemic samples, enabling a broader understanding of SARS-CoV-2-induced alterations in the RAAS profile. The analysis was designed to identify biochemical indicators that could help to understand the mechanisms of convalescence and the basis of the occurrence of long COVID syndrome, including the impact of SARS-CoV-2 infection on the possible progression of pathological changes in the cardiovascular system, e.g., arterial hypertension.

## 2. Materials and Methods

### 2.1. Blood Sample Collection

The study was performed in accordance with the guidelines of the Declaration of Helsinki, and ethical approvals were obtained from the Bioethics Committee at Wroclaw Medical University (KB 536/2022 approved on 27 July 2022 and KB 253/2024 approved on 18 April 2024). Additionally, the use of archived serum samples and anonymized demographic data was granted by the Directorate of the Regional Center for Blood Donation and Blood Treatment in Wrocław (approval date: 1 July 2022).

Samples were collected from participants who met inclusion criteria defined by European guidelines, including those issued by the European Commission Directorate-General for Health and Food Safety on 4 April 2020. These guidelines were developed by the team of the National Consultant for Transfusiology and endorsed by the Public Blood Service Continuity Management Working Group [19]. The key recommendations are summarized below:

- A confirmed past SARS-CoV-2 infection (positive result of RT-PCR test);
- Good health at the time of each donation (no symptoms of COVID-19 or any other disease and not a carrier);
- Fulfillment of standard eligibility criteria for blood donation, including age, body weight, blood pressure, heart rate, and hemoglobin level, as defined by the regulation of the Minister of Health.

A total of 394 convalescent plasma donors who had donated plasma at least twice following confirmed SARS-CoV-2 infection in 2020–2021 were initially recruited. From this group, 30 individuals were selected for the final study cohort. Each of these donors had donated plasma four times at regular intervals of 30 ± 2 days, resulting in a total of 120 analyzed samples. Based on this sampling schedule, four time periods were defined for analysis: Period 1: ≤60 days; Period 2: 61–90 days; Period 3: 91–120 days; Period 4: >120 days.

As a control group, archived serum samples collected from 30 regular blood and blood component donors in the pre-pandemic period (2018–2019) were used.

Control group selection criteria, including age and sex, were matched to those of participants in the study group (*p* > 0.05).

Control and experimental blood samples were collected in BD Vacutainer SST II Advance serum tubes with separating gel (Becton Dickinson, Plymouth, UK) and subsequently centrifuged at 3000 rpm for 5 min. The resulting supernatant was transferred into 1.5 mL polypropylene tubes and stored at −20 °C without being subjected to any freeze–thaw cycles. This method of sample collection is standard practice during each donation, as it is required for routine hematological and biochemical testing prior to blood or plasma donation.

### 2.2. RAAS Parameter Determination

ELISA test kits were used to determine selected parameters of the RAAS, such as Ang I, Ang II, Ang 1–9, Ang 1–7, ACE, ACE2, ADAM10 and ADAM17 (Shanghai Sunred Biological Technology Co., Ltd., Shanghai, China). The sensitivity of the Ang I assay was 3.658 ng/L, that of the Ang II assay was 1.352 pg/mL, that of the Ang 1–9 assay was 7.241 ng/L, that of the Ang 1–7 assay was 53.113 ng/L, that of the ACE assay was 0.123 ng/mL, that of the ACE2 assay was 0.368 ng/mL, that of the ADAM10 assay was 0.088 ng/mL, and that of the ADAM17 assay was 0.676 ng/mL. The measurements were performed in triplicate in accordance with the manufacturer’s instructions. Briefly, 40 µL of serum samples were added to microplate wells pre-coated with specific monoclonal antibodies. Then, 10 µL of biotin-conjugated specific antibodies and 50 µL of HRP-labeled streptavidin were added to each well, and the plates were incubated at 37 °C for 1 h with gentle mixing. After washing, substrate solution was added, and the plates were incubated in darkness with gentle mixing. The reaction was stopped after 10 min. Absorbance was measured at 450 nm using a microplate reader (model 800 TS, BioTek Instruments, Winooski, VT, USA).

### 2.3. Statistical Analysis

The distribution of the data was tested with the Shapiro–Wilk normality test. Descriptive data were presented as the number and percentages or the median and interquartile range (IQR). The Mann–Whitney test was used for analysis of differences between two independent groups. Friedman’s test was performed for comparison of data between more than two dependent samples, and the paired-samples sign test was used as a post hoc test. Single correlations were analyzed by Spearman’s rank correlation test. All values of *p* ≤ 0.05 were assumed to be statistically significant. Data were analyzed using Statistica v. 13.3 (Tibco Software Inc., Palo Alto, CA, USA).

## 3. Results

In both the study and control groups (n = 30), the majority (over 80%) were men, which is consistent with the general trend among donors of blood and its components, who are mainly men. The median age in the control group was 37 [IQR: 34–42], and in participants in the study group, it was 44 [IQR: 32–47] years (*p* = 0.226). The complete dataset has been presented in our previously published paper [20].

Table 1 presents the concentrations of the analyzed parameters in the control group and in the study group across four defined time periods. Our findings demonstrated that in Period 1, parameters such as ACE2, Ang 1–9, and Ang 1–7 were significantly higher than in the control group (for ACE 2: 48.16 ng/mL vs. 28.36 ng/mL, *p* = 0.014; for Ang 1–9: 1150.54 ng/L vs. 701.31 ng/L, *p* = 0.018; and for Ang 1–7: 488.00 ng/L vs. 397.00 ng/L, *p* = 0.021, respectively). The data for Period 2 showed statistically significant differences only for Ang 1–7 in control vs. Ang 1–7 in Period 2, *p* = 0.049.

**Figure 1:**
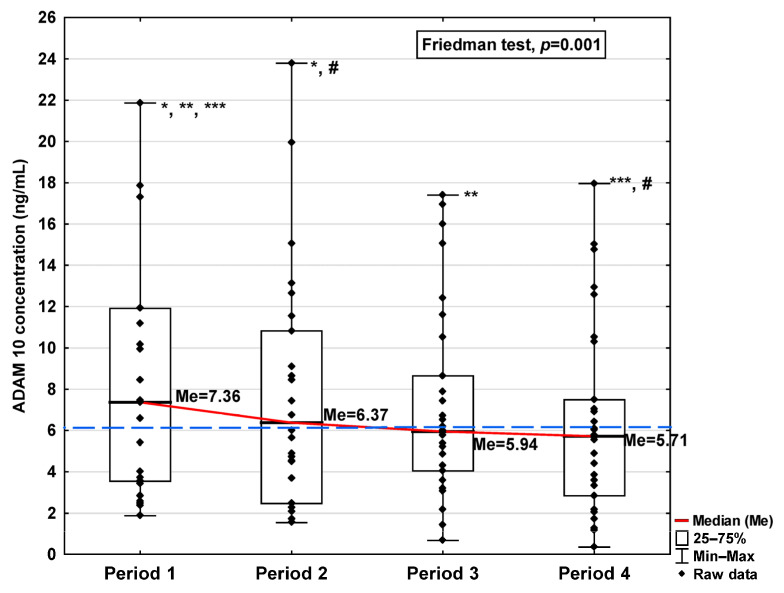
Serum concentration of ADAM 10 in the blood donors in Periods 1–4 after SARS-CoV-2 infection. The red line shows median kinetics in Periods 1–4; the blue horizontal dashed line indicates the median of the ADAM 10 concentration in the control group. *: Period 1 vs. Period 2, *p* = 0.017; **: Period 1 vs. Period 3, *p* = 0.044; ***: Period 1 vs. Period 4, *p* = 0.018; #: Period 2 vs. Period 4, *p* = 0.018.

**Figure 2:**
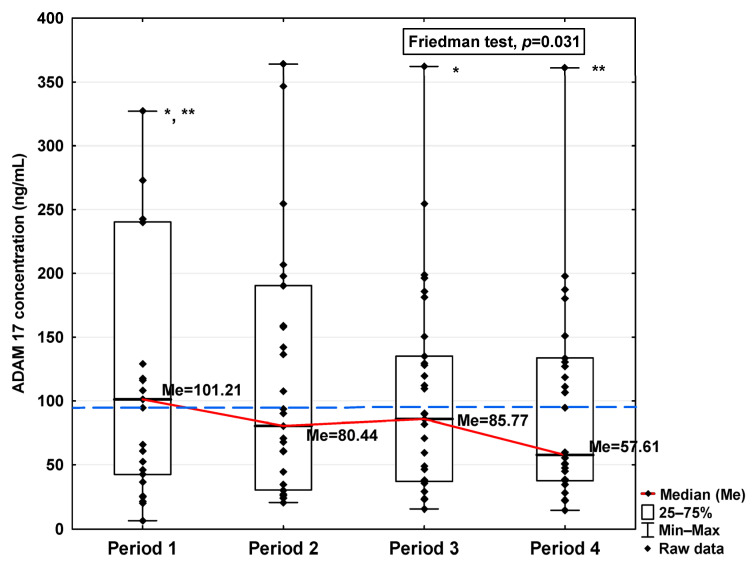
Serum concentration of ADAM 17 in the blood donors in Periods 1–4 after SARS-CoV-2 infection. The red line shows median kinetics in Periods 1–4; the blue horizontal dashed line indicates the median of the ADAM 17 concentration in the control group. *: Period 1 vs. Period 3, *p* = 0.044; **: Period 1 vs. Period 4, *p* = 0.034.

**Figure 3:**
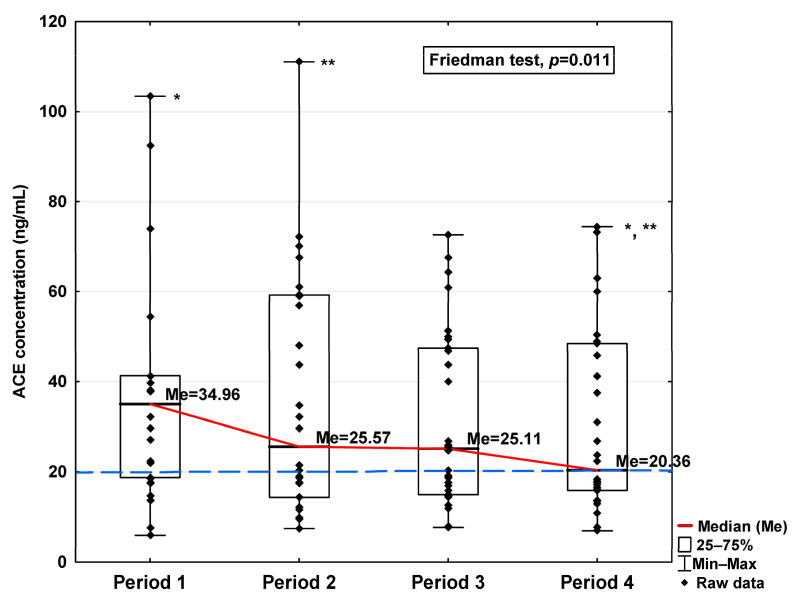
Serum concentration of ACE in the blood donors in Periods 1–4 after SARS-CoV-2 infection. The red line shows median kinetics in Periods 1–4; the blue horizontal dashed line indicates the median of the ACE concentration in the control group. *: Period 1 vs. Period 4, *p* = 0.045; **: Period 2 vs. Period 4, *p* = 0.044.

**Figure 4:**
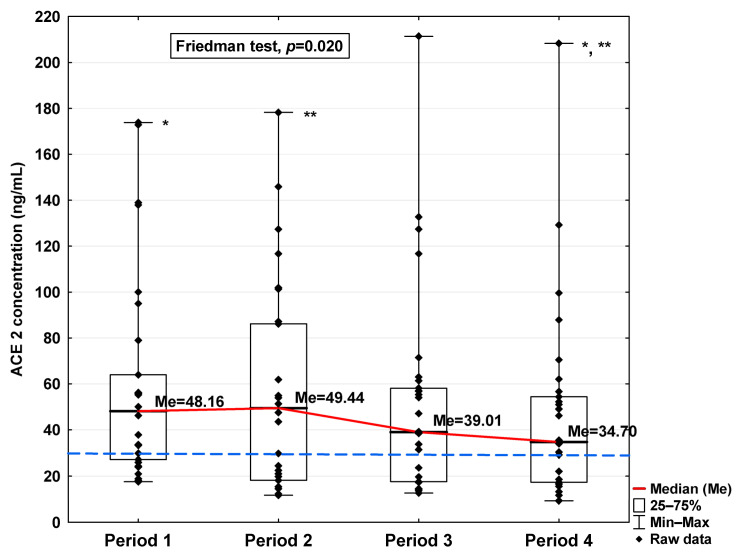
Serum concentration of ACE2 in the blood donors in Periods 1–4 after SARS-CoV-2 infection. The red line shows median kinetics in Periods 1–4; the blue horizontal dashed line indicates the median of the ACE2 concentration in the control group. *: Period 1 vs. Period 4, *p* = 0.045; **: Period 2 vs. Period 4, *p* < 0.001.

**Figure 5:**
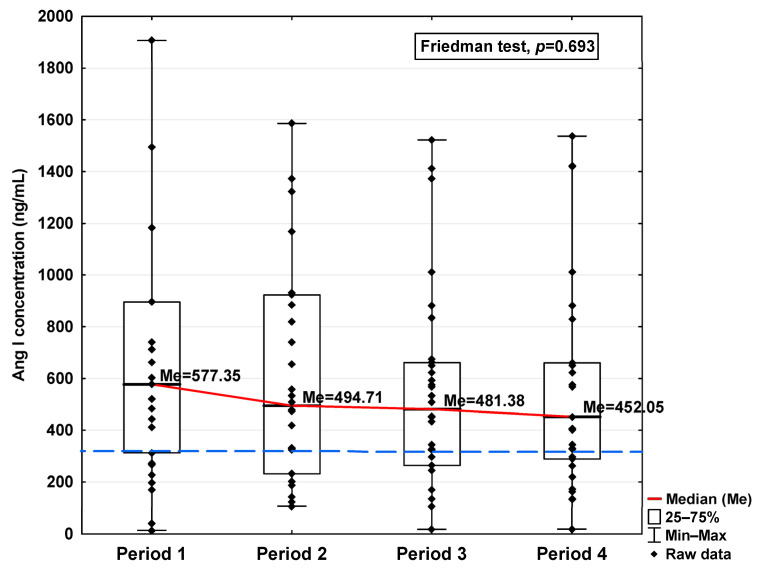
Serum concentration of Ang I in the blood donors in Periods 1–4 after SARS-CoV-2 infection. The red line shows median kinetics in Periods 1–4; the blue horizontal dashed line indicates the median of the Ang I concentration in the control group.

**Figure 6:**
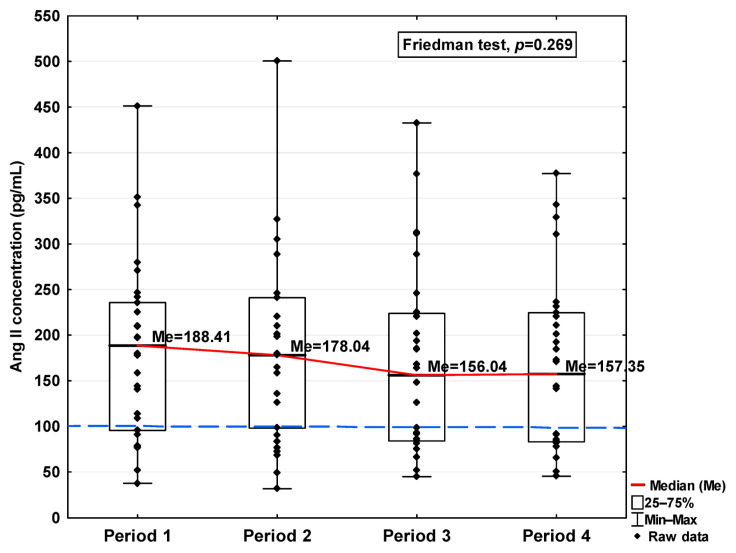
Serum concentration of Ang II in the blood donors in Periods 1–4 after SARS-CoV-2 infection. The red line shows median kinetics in Periods 1–4; the blue horizontal dashed line indicates the median of the Ang II concentration in the control group.

**Figure 7:**
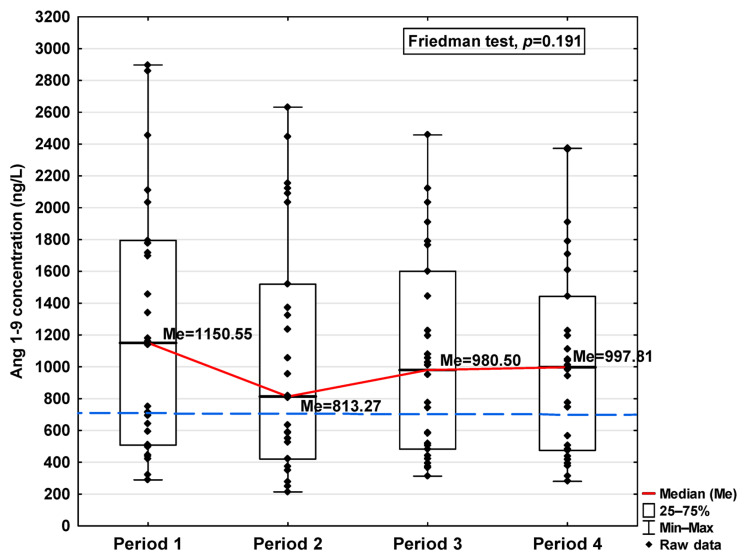
Serum concentration of Ang 1–9 in the blood donors in Periods 1–4 after SARS-CoV-2 infection. The red line shows median kinetics in Periods 1–4; the blue horizontal dashed line indicates the median of the Ang 1–9 concentration in the control group.

**Figure 8:**
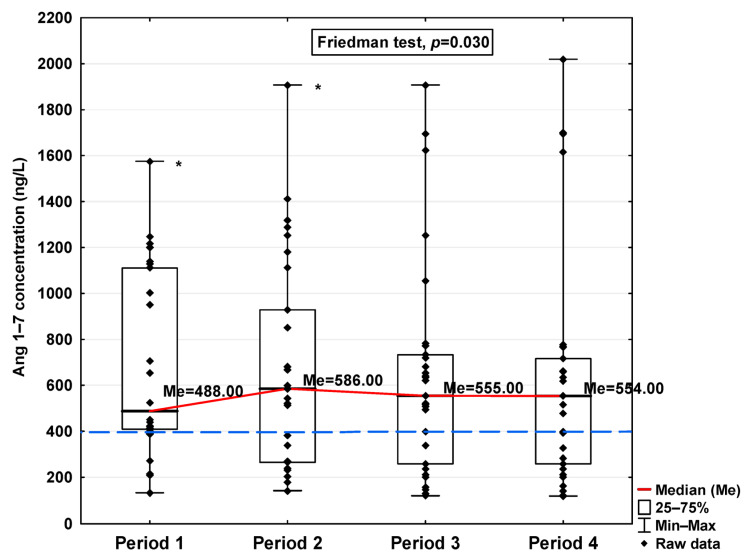
Serum concentration of Ang 1–7 in the blood donors in Periods 1–4 after SARS-CoV-2 infection. The red line shows median kinetics in Periods 1–4; the blue horizontal dashed line indicates the median of the Ang 1–7 concentration in the control group. *: Period 1 vs. Period 2, *p* < 0.001.

In Figure 1, Figure 2, Figure 3, Figure 4, Figure 5, Figure 6, Figure 7 and Figure 8, results obtained for each of the eight parameters studied are presented. The dashed line indicates the median of the results obtained for the control. We also mark statistically significant differences obtained in the individual study periods.

The obtained ADAM10 concentration data indicate that SARS-CoV-2 infection causes, in the initial period after infection, an increase in the concentration of this parameter (7.36 ng/mL in Period 1 vs. 6.14 ng/mL in control group; Table 1), dropping to values slightly lower than for the control after 120 days from infection (5.71 ng/mL). We can also observe that the level of ADAM10 was significantly higher in Period 1 compared to Periods 2, 3, and 4 (*p* = 0.017, *p* = 0.044, and *p* = 0.018, respectively; Figure 1), and that the level in Period 2 was significantly higher than in Period 4 (*p* = 0.018). The ADAM17 concentration shows a similar trend, also increasing in Period 1 (101.21 ng/mL vs. 87.50 ng/mL in control group; Table 1), dropping to a lower level than the control in Period 4 (57.61 ng/mL), although this is still not a statistically significant decrease. We also detected higher concentrations of ADAM17 in Period 1 than in Periods 3 and 4 (*p* = 0.044 and *p* = 0.034, respectively; Figure 2).

Regarding angiotensin-converting enzymes ACE and ACE2, we observe an increase in these indicators in the initial period after infection (34.96 ng/mL vs. 20.25 ng/mL and 48.16 ng/mL vs. 28.36 ng/mL, respectively; Table 1), finding a decrease in subsequent periods to a level similar to the concentration in the control (20.36 ng/mL and 34.70 ng/mL, respectively). The ACE concentration in Period 1 was significantly higher compared to Periods 2 and 4 (*p* = 0.045 and *p* = 0.044, respectively; Figure 3). A significantly higher ACE2 concentration was observed in the first period studied compared to the control (*p* = 0.014; Table 1), but it was lower in Period 1 than in Period 2 (*p* = 0.045; Figure 4). We also determined that in Period 2, its concentration was higher than in Period 4 (*p* < 0.001; Figure 4).

The concentrations of Ang I and Ang II also increased in the first period studied compared to the control (577.35 ng/mL vs. 335.76 ng/mL and 188.41 pg/mL vs. 100.78 pg/mL, respectively; Table 1), but while they decreased in the subsequent periods studied, even in Period 4 they were higher than the level observed in the case of the control (452.05 ng/mL and 157.35 pg/mL, respectively, Table 1). Although variations were observed, no statistically significant differences in either of these parameters were found across the analyzed time periods (Figure 5 and Figure 6).

Regarding Ang 1–9 and Ang 1–7, a statistically significant increase in the concentration of these parameters was observed in Period 1 compared to the control (1150.54 ng/L vs. 701.31 ng/L and 488.00 ng/L vs. 397 ng/L, respectively; Table 1). In the subsequent periods, the concentration decreased, but also, even in Period 4, it remained at a higher level than in the control group (997.81 ng/L and 554.00 ng/L, respectively). While no significant differences in Ang 1–9 concentrations were observed between the study periods (Figure 7), Ang 1–7 levels were found to be significantly lower in Period 1 compared to Period 2 (*p* < 0.001; Figure 8).

**Figure 9:**
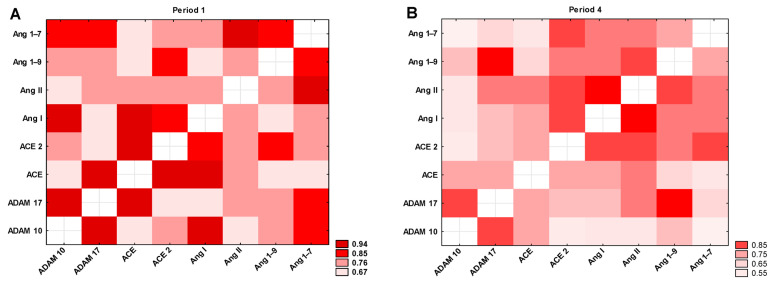
Heatmap of correlation coefficients of selected variables in (**A**) Period 1 and (**B**) Period 4. All colors indicate a statistically significant correlation between the tested parameters (*p* < 0.0001).

The heatmap (Figure 9A) shows a comparison of the mutual correlations between the tested parameters in Period 1. All correlation coefficients between the tested parameters were statistically significant: r = 0.66–0.94, *p* < 0.0001. The highest positive correlation is seen in the case of ADAM10 vs. ADAM17 (r = 0.93), followed by Ang II vs. Ang 1–7 (r = 0.91), ADAM10 vs. Ang I, ADAM17 vs. ACE, and ACE vs. ACE2 (in all cases r = 0.89), and ACE vs. Ang I (r = 0.87). Since our study aimed to investigate long-term changes, we analyzed correlations among the parameters measured in Period 4. The results showed a strong positive correlation between ADAM10 and ADAM17 (r = 0.81), ADAM17 and Ang 1–9 (r = 0.89), and Ang I and Ang II (r = 0.85). All correlation coefficients between the tested parameters were statistically significant (r = 0.51–0.89; *p* < 0.0001; Figure 9B).

## 4. Discussion

Currently, the spread of SARS-CoV-2 is effectively limited by the use of widespread vaccinations in the population and also by implementing the recommended COVID-19 therapies, such as antiviral drugs or immunomodulatory therapies [21]. Nevertheless, a significant number of patients continue to experience long-term health consequences associated with COVID-19. Nonspecific symptoms of long COVID—such as fatigue, myalgia, palpitations, cognitive impairment, dyspnea, anxiety, and chest and joint pain—have been linked to various metabolic disturbances induced by SARS-CoV-2, including dysbiosis, immune dysregulation, and thrombosis [22,23,24].

SARS-CoV-2 infection leads to a reduction in membrane-bound ACE2 (mACE2) due to the direct effects of the virus [25,26]. Concurrently, an increase in soluble ACE2 (sACE2) levels is observed [27], which may play a compensatory role in response to imbalances in the RAAS. These changes are part of the body’s complex response to infection, explained by the fact that increased sACE2 levels may protect by converting angiotensin II (Ang II) to angiotensin 1–7, which has vasodilatory and anti-inflammatory effects [28,29,30]. In our study, only sACE2 levels could be assessed due to the use of serum samples. In contrast, studies based on tissue homogenates may reflect both mACE2 and sACE2 contents.

We found statistically significant positive correlations between ACE and ACE2, ACE and Ang I, and Ang II and Ang 1–7, consistent with the known enzymatic activity of ACE and ACE2. Additionally, we observed significant increases in ACE and ACE2 levels within 60 days post-infection, potentially accounting for the elevated concentrations of their downstream products. Importantly, although Ang 1–9 and Ang 1–7 levels decreased over time, they remained higher than in the control group even 120 days post-infection. The elevated level of ADAM17 may explain the increased concentration of sACE2. From a long-term perspective, we observed a decrease in the concentrations of parameters such as ADAM10, ADAM17, and Ang 1–9; however, the latter remained elevated compared to the control group. This may suggest an initial pro-inflammatory and profibrotic effect of the metalloproteinases, which over time becomes counterbalanced by the action of Ang 1–9, a molecule with anti-inflammatory properties. This observation highlights the complex nature of compensatory mechanisms within the renin–angiotensin system.

The long-term observation of the tested parameters, which extended beyond 120 days post-infection, showed that most values returned to physiological ranges observed in the control group. Despite statistically significant fluctuations in some parameters across time points, none of the participants reported clinical symptoms or hematological abnormalities that would disqualify them from further blood donation. These findings are consistent with our previous reports [20,31].

We also observed that ADAM10 and ADAM17 levels were elevated shortly after infection but decreased below control levels by the final time point (day 120). Positive correlations were found between ADAM10 and ADAM17, ADAM17 and ACE, and ADAM10 and Ang I. According to Zipeto et al., SARS-CoV-2 can induce a robust inflammatory response in which ADAM17 plays a key role by releasing pro-inflammatory cytokines such as TNF-α and their receptors, potentially exacerbating cardiovascular dysfunction. Increased ADAM17 activity may also lead to ACE2 shedding, disrupting the Ang II/Ang 1–7 balance, which may contribute to elevated blood pressure and inflammation [32].

The increase in both metalloproteinases in the early post-infection period observed by us is also consistent with the reports of Josher et al. on the influence of ADAM10 and ADAM17 on facilitating the entry of SARS-CoV-2 into host cells [33]. Lartey et al. also suggested that ADAM17 inhibition reduced neutrophilia and lung damage in a rat model, which could be used in the treatment of severe pulmonary complications of COVID-19 [34]. In addition, Sun et al. showed a positive correlation between ADAM17 and ACE2 and additionally linked it to the severity of COVID-19 [35]. A reduction in pulmonary mortality was also demonstrated after SARS-CoV-2 infection in mice after knocking out the ADAM17 gene [35] or after neutralizing this metalloproteinase with antibodies [36].

ADAM17 is known to be involved in the proteolytic cleavage of precursors such as Ang I, facilitating the formation of active products like Ang II [10]. Elevated ADAM17 levels may therefore enhance RAAS activity, particularly in response to SARS-CoV-2-induced inflammation. Ang II is a potent pro-inflammatory mediator and regulator of blood pressure, with a key role in the cardiovascular complications of COVID-19. Ang II and its metabolites, including Ang 1–7, have been shown to modulate inflammatory responses [11,30]. Excess Ang II may trigger exaggerated immune activation and inflammation, particularly during the acute phase of infection. ADAM17 also influences cytokine receptor activity, potentially amplifying the inflammatory response [37]. Dysregulation of the ACE/ACE2 axis by ADAM17 may further impact vascular tone and fluid homeostasis, contributing to complications such as pulmonary hypertension and organ damage in severe COVID-19.

Taken together, the observed long-term alterations in RAAS parameters, particularly elevated sACE2 levels and dysregulated angiotensin peptide profiles, may have relevant clinical implications. Persistent RAAS imbalance can potentially impair vascular tone regulation, contribute to blood pressure variability, and increase the risk of endothelial dysfunction [7,38]. Chronically elevated ACE2 may reflect ongoing compensatory mechanisms, yet insufficient conversion of Ang II to Ang 1–7 could favor pro-inflammatory and profibrotic pathways [38]. This may predispose convalescent individuals to cardiovascular complications, such as post-viral myocarditis, arrhythmias, or hypertension [7]. In the renal context, dysregulated RAAS could affect sodium and water handling, potentially leading to volume dysregulation or contributing to proteinuria. In the pulmonary system, RAAS imbalance and persistent ADAM17 activity might play a role in post-COVID lung remodeling or fibrosis, especially in patients with pre-existing conditions [39,40]. These findings underscore the need for longitudinal monitoring of RAAS-related biomarkers in recovered individuals, particularly those with symptoms consistent with long COVID.

### Limitations of the Study

One limitation of the study is the predominance of male participants. This reflects the demographic characteristics of repeat blood donors in Poland, where men constitute the majority due to donation frequency regulations and eligibility criteria. Although this sex imbalance may limit the generalizability of the findings to female populations, it also helped reduce variability related to hormonal fluctuations that can influence RAAS activity.

Another limiting factor of the study is that the samples were collected from a limited number of individuals. This was a consequence of the stringent criteria we applied to ensure greater data reliability. Specifically, we aimed to include only those participants who donated plasma in all four study periods, allowing for intra-individual comparisons. However, it is uncommon for donors to return to the blood donation center at regular intervals, which significantly reduced the available pool of eligible participants. Another limitation is the scope of available clinical data. During donor qualification, only a standard medical interview related to blood donation was conducted. No additional clinical data were collected regarding the course of COVID-19, and thus we cannot provide information on symptom severity. In future studies, including a targeted interview focusing on symptoms experienced during the acute phase of infection would allow for correlation with biochemical parameters. Moreover, we had no access to follow-up information confirming or excluding potential SARS-CoV-2 reinfections among donors, so reinfection status could not be accounted for in the analysis.

## 5. Conclusions

In our study, elevated levels of the described parameters were primarily observed during the initial phase following infection. Subsequently, the parameters returned to baseline, which may explain the absence of symptoms or deterioration in well-being among the participants. Their good overall health also allowed them to donate blood and its components in accordance with current eligibility guidelines [19].

Although the increases in ADAM10 and ADAM17 in Period 4 did not reach statistical significance, their mutual correlation, along with a positive association with the alternative RAAS pathway (reflected by elevated Ang 1–9 levels), may suggest a trend toward regulatory shifts. Over time, such changes could potentially contribute to vascular remodeling processes, including fibrosis, although this hypothesis requires further study.

Thus, while ADAM10, ADAM17, Ang I, and Ang II may play a role in the pathophysiology of COVID-19, the lack of symptom progression in our participants underscores the need for continued research into the biochemical impact of SARS-CoV-2-induced dysregulation of RAAS, not only in blood but also across cardiovascular, renal, and pulmonary systems.

## Figures and Tables

**Table 1:** Comparison of the serum concentrations of tested parameters between healthy control individuals and repeat blood donors in Periods 1–4 after SARS-CoV-2 infection. Data are presented as median [IQR], and differences between two independent groups were calculated with the Mann–Whitney test.

	Control Group (n = 30)	Period 1 (n = 30)	Period 2 (n = 30)	Period 3 (n = 30)	Period 4 (n = 30)
ADAM 10 (ng/mL)	6.14 [4.00–9.11]	7.36 [3.53–11.91]	6.37 [2.47–10.82]	5.94 [4.03–8.63]	5.71 [2.84–7.49]
ADAM 17 (ng/mL)	87.50 [54.53–136.38]	101.21 [42.35–240.38]	80.44 [30.11–190.51]	85.77 [36.80–135.25]	57.61 [37.35–133.80]
ACE (ng/mL)	20.25 [12.65–48.50]	34.96 [18.72–41.25]	25.57 [14.31–59.22]	25.11 [14.90–47.40]	20.36 [15.87–48.40]
ACE 2 (ng/mL)	28.36 [14.15–54.61]	48.16 [27.20–63.97] *	49.44 [18.16–86.17]	39.01 [17.61–58.10]	34.70 [17.35–54.41]
Ang I (ng/mL)	335.76 [210.38–696.53]	577.35 [312.64–895.58]	494.71 [230.88–923.23]	481.38 [263.82–661.47]	452.05 [288.52–660.47]
Ang II (pg/mL)	100.78 [51.05–228.94]	188.41 [95.54–235.83]	178.04 [98.19–240.98]	156.04 [84.04–223.70]	157.35 [83.04–224.51]
Ang 1–9 (ng/L)	701.31 [435.53–970.15]	1150.54 [507.81–1795.09] **	813.27 [420.54–1519.63]	980.50 [483.27–1601.50]	997.81 [475.09–1442.27]
Ang 1–7 (ng/L)	397.00 [238.78–528.07]	488.00 [409.33–1111.33] ***	586.00 [265.33–928.00] ****	555.00 [259.33–732.80]	554.00 [258.34–716.67]

*: ACE 2 in control vs. ACE 2 in Period 1, *p* = 0.014; **: Ang 1–9 in control vs. Ang 1–9 in Period 1, *p* = 0.018; ***: Ang 1–7 in control vs. Ang 1–7 in Period 1, *p* = 0.021; ****: Ang 1–7 in control vs. Ang 1–7 in Period 2, *p* = 0.049.

## Data Availability

The data are available from the corresponding author and may be shared if necessary.
